# Superior residual fertiliser value in soil with phosphorus recycled from urine in layered double hydroxides

**DOI:** 10.1038/s41598-022-11892-4

**Published:** 2022-05-16

**Authors:** K. Dox, T. Martin, S. Houot, R. Merckx, E. Smolders

**Affiliations:** 1 Division of Soil and Water Management, Department of Earth and Environmental Science, KU Leuven, Kasteelpark Arenberg 20, 3001 Heverlee, Belgium; 2 UMR ECOSYS, INRAE, AgroParisTech, Université Paris-Saclay, Avenue Lucien Bretignières, 78850 Thiverval-Grignon, France

**Keywords:** Biogeochemistry, Environmental sciences, Nanoscience and technology

## Abstract

Layered double hydroxides (LDHs) of magnesium (Mg) and aluminium (Al) are ion exchangers that can be used as slow release phosphorus (P) fertilisers. These LDHs can be used successfully to concentrate P from waste streams such as urine. This study was set up to test the fertiliser potential of P derived from urine and concentrated on LDHs. Ryegrass was grown in a pot trial using a P- and N-deficient soil where different urine derived fertilisers, i.e. LDH-P, stored urine and urine mixed with sludge as a source of P were compared to different mineral N and P doses in a full factorial design. Plants were grown for 75 days with four cuttings and did not exhibit salinity stress in stored urine treatments. Plant growth and P uptake responded to N, P doses in mineral fertilizer treatments with significant N-P interaction. The fertiliser use efficiency of urine fertilisers was lower than that of mineral fertilisers at equivalent total nutrient input for stored urine, due to lower N availability, and for urine mixed with sludge due to lower P availability. In contrast, the yield and P uptake of ryegrass grown on LDH loaded with P from urine (LDH-P) showed equal fertiliser P use as mineral fertiliser. Interestingly, the residual soil P after harvest, scored by the sum of isotopically exchangeable P in soil and the P uptake, was higher for LDH-P than for mineral P, confirming slow release properties of LDH that limit loss of P by fixation in soil.

## Introduction

Large amounts of phosphorus (P) are applied in agriculture as fertilisers to maintain crop yields. Soluble P fertilisers need to be dosed in excess of crop requirements in several soils because of low P fertiliser use efficiency (PUE), this is especially in acid weathered tropical soils^1^ or in highly calcareous soils^2^. In acid weathered soils, P is sorbed strongly onto Fe and Al oxyhydroxides^3^ that are abundant in these soils. In contrast, in calcareous soils part of added P becomes unavailable due to precipitation as insoluble calcium phosphates. The P fertilisers are mainly produced from rock phosphate, a finite natural resource^4^. The estimations of rock phosphate reserves vary widely, but rock phosphate is eventually limited, necessitating the need for sustainable alternatives and increased P recycling.

Fertilisers derived from recycled P hold large potential in closing the P cycle. The recycling of P is hampered by the potential to concentrate the soluble P into a fertiliser with high PUE. Urine is a well-defined waste stream that mainly contains anionic phosphate (denoted here as PO_4_ but mainly present as the H_2_PO_4_^-^ at the pH of urine). Global human urine P emissions are 2 Mt P/annum^5^, that is about 14% of the total annual use of P in mineral P fertilisers^6^. More than 50% of the total P in domestic waste water is derived from urine^7^. The primary technology to remove P from wastewater is the addition of iron (Fe) and aluminium (Al) salts resulting in precipitation of FePO_4_ and AlPO_4_ in the sludge^8^. This technology is focused on P removal from solution to lower the water eutrophication risks rather than on P recycling. This is because the PUE of FePO_4_ and AlPO_4_ precipitates in the sludge is very low^9^.

Removal of P from wastewater can also be achieved by biological P removal. Here, much less sludge is produced and the use of chemicals is avoided^8^. An additional benefit of biological wastewater treatment is that the reactors can be designed to remove multiple nutrients plus biological oxygen demand simultaneously^10^. Furthermore, the P retained is much more bioavailable than the P in precipitated sludge and can be used as efficient P fertiliser after treatment^11,12^.

An emerging technology is the precipitation of struvite (MgNH_4_PO_4_·6H_2_O)^7,13,14^. The addition of magnesium (Mg) results in oversaturation which induces the precipitation. Struvite precipitation removes both nitrogen (N) and P simultaneously. When no ammonium is present in the wastewater, a variation of the technology can be used by precipitating MgKPO_4_·6H_2_O^15,16^. While impurities are inserted in the struvite crystals during precipitation^7^, the end-product is much purer than in heterogeneous biological sludge. Furthermore, multiple researchers reported a high agronomical potential of struvite^11,17–19^. Furthermore, struvite could have slow-release properties^19^, which could increase yields in the long term.

A new method for P recycling from urine is based on the use of layered double hydroxides (LDHs) as an ion exchange material that reversibly bind PO_4_^20^. The LDHs are a class of anionic clay minerals with the general chemical formulas of $\left[ {{\text{M}}^{{{2} + }}_{{{1} - {\text{x}}}} {\text{M}}^{{{3} + }}_{{\text{x}}} \left( {{\text{OH}}} \right)_{{2}} } \right]^{{{\text{x}} + }} \left[ {{\text{A}}^{{{\text{n}} - }} } \right]_{{{\text{x}}/{\text{n}}}} \cdot{\text{yH}}_{{2}} {\text{O}}\;{\text{ or}}\; \, \left[ {{\text{LiAl}}_{{2}} \left( {{\text{OH}}} \right)_{{6}} } \right]^{ + } \left[ {{\text{A}}^{{{\text{n}} - }} } \right]_{{{1}/{\text{n}}}} \cdot{\text{yH}}_{{2}} {\text{O}}$^21^. Synthesis of these materials is relatively easy and cheap and can be tuned for different technologies. Recently, we demonstrated that LDHs are efficient ion exchange materials for P in urine and that a high P removal efficiency can be reached^20^.

Phosphate-loaded LDH are efficient P fertilisers^22–24^ showed an equal P fertiliser efficiency of struvite and P-loaded LDHs. Furthermore, just like struvite, P-loaded LDHs could have slow release-properties, potentially increasing long-term yields^22,23^. In calcareous soils, LDH-P may theoretically have advantages over struvite P on the residual P fertiliser value (long-term fertiliser value) because LDH-P is likely released at lower concentrations in solution than the soluble struvite, thereby avoiding formation of Ca-phosphates.

The fertiliser values of LDH-P have been evaluated in recent studies; however no comparative study was yet made on the PUE of LDH-P when recycled from urine. Therefore, this study was made to compare the relative PUE of different urine P derived P fertilisers, including the LDHs. Urine is a source of P and N and, therefore, any comparison with stored urine requires a multivariate testing of P and N fertiliser potential. This was evaluated with a N and P deficient pot trial and fertilisers included P-loaded LDH, stored urine and urine mixed with sludge. The PUE was referenced to treatments including mineral N and P doses and urine fertilisers supplemented with mineral N- and P. To better evaluate the potential long term benefits, residual P fertiliser values were estimated using isotopically exchangeable P in soil after harvest. This study was part of a larger program of urine based fertilisers and the nitrogen availability in urine was tested with multiple additional treatments, these data are communicated in a different paper while the study here focuses on the P related aspects only. Effects of the urine doses on plant cadmium (Cd) availability are reported here since chloride salinity is well known to enhance soil Cd bioavailability^25^.

## Materials and methods

### Soil

A N&P deficient arable soil was sampled in the plough layer of a silty luvisol at the Folleville field trial located in Thiverval-Grignon (France) in April 2018. The soil was sampled from plots under arable land that had not received any mineral or organic fertilization since 1986 but was continuously cropped. The pH (0.01 M CaCl_2_) is 6.7, the cation exchange capacity (Cobaltihexamine extraction /ISO 23,470) is 17.9 cmo_c_/kg, mineral soil N was 11.9 mg N/kg dry soil, acid ammonium oxalate-P concentration was 240 mg/kg and the 0.01 M CaCl_2_ extractable P was 0.24 mg/kg, the latter two values indicate low to deficient P supply as scored by thresholds derived in field trials^26^.

### Fertilisers

The urine was recovered during spring 2018 using a dry male urinal installed at École des Ponts et Chaussées Paris Tech. The urine was stored for few months in closed tanks before the experiment. Urine often has to be stored before it can be used. The total N concentration in the urine is 6.99 g N/L. During storage about 30% of phosphorus precipitated at the bottom of the tank. The precipitated P was not used during the experiment. The P concentration in the urine at the time of the experiment was 0.58 g/L.

The sewage sludge comes from a multistep water treatment plant in France and is as such the product of both chemical and biological P removal. The P concentration in the sludge is 17.86 mg P/g dry sludge and the N concentration is 9.1 mg N/g dry sludge. Importantly, as a result of the precipitation step, the concentrations of Fe and Al are high (98.5 mg Fe/g dry sludge and 9.5 mg Al/g dry sludge).

The MgAl LDHs were synthesised using the coprecipitation method^27^. A precursor solution was prepared by mixing 2 M of Mg(NO_3_)_2_·6H_2_O with 1 M of Al(NO_3_)_3_·9H_2_O and 50 mL of this precursor solution is added dropwise over 5 h using a perfusor pump (Braum Perfusor Space) to 400 mL of continuously stirred ultrapure water. During precipitation, the reactor vessel was flushed with N_2_ gas to remove CO_2_, thus minimizing CO_3_^2−^ from the precipitation solution. The pH of the precipitation solution was kept constant at pH 10 by controlled addition of a 3 M NaOH solution using a titrator (Metrohm 702SM Titrino). The LDH slurry was then recovered by centrifugation (1000 g, 20 min), washed twice by resuspension and dried at 60 °C. The dried LDHs were exchanged with PO_4_ by shaking them end-over-end in synthetic urine^28^ at a pH of 6 and a solid liquid ratio of 5 g LDH/L for 5 h. Again, the P-loaded LDHs (LDH-P) are recovered by centrifugation (10,000 g, 20 min) and dried at 60 °C. The P content of the LDH-P was measured by dissolution in nitric acid followed by P analysis with Inductively Coupled Plasma-Optical Emission Spectroscopy (ICP-OES, Thermo Scientific, iCAP 7000 series) and, for Cd, with Inductively Coupled Plasma-Mass Spectrometry (ICP-MS, Agilent technologies, 7700 series). Physicochemical analysis of the material has been discussed in previous research^20^*.*

### Experimental design

**Figure 1:**
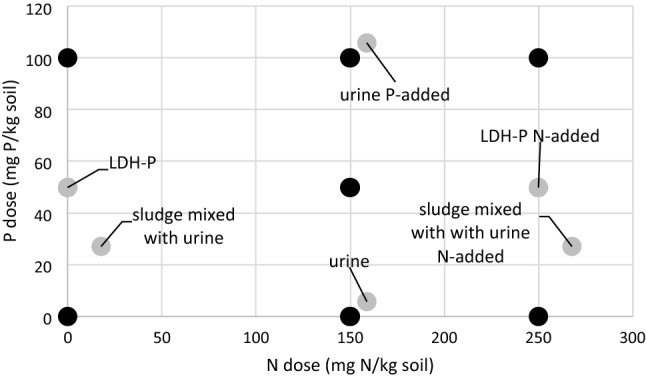
Experimental design of the various fertiliser treatments: the P doses plotted versus N dose. The black circles show the doses of the mineral treatments using NH_4_NO_3_ for mineral N and KH_2_PO_4_ form mineral P. The grey circles are urine derived fertilisers, the hatched grey are showing where mineral treatments (black) overlap with urine derived treatments.

Ryegrass was grown in a pot trail using a P- and N-deficient soil with 15 different fertiliser treatments (Fig. 1). These 15 treatments included nine mineral N and P fertilizer treatments in a 3 × 3 factorial design of N and P doses, i.e. 0, 150 and 250 mg N/kg soil (as NH_4_NO_3_) or 0, 50 and 100 mg P/kg soil (as KH_2_PO_4_) in all combination. These nine treatments refer to fully soluble fertilisers and are the reference for six different urine derived fertilisers, i.e. LDH-P (with or without added mineral N), stored urine (with or without mineral P) and urine mixed with sludge (with or without added mineral N). Urine has a N/P ratio above plant N/P and, therefore, needs P addition for a balance fertilisation. Conversely, sewage sludge N/P ratio is high and needs mineral N addition for balancing the supply. The research conducted complied with all relevant institutional as well as regional, national and international guidelines and legislation.

### Plant growth

Ryegrass was grown in a pot trial in the greenhouse the 15 different treatments (Fig. 1), each treatment had three replicates of 1.17 kg potted dry soil in a 1 L pot. The mineral N fertilisers added 0, 150 and 250 mg N/kg as NH_4_NO_3_ while P fertiliser added 0, 50 and 250 mg P/kg as KH_2_PO_4_ in a full factorial design. In the urine treatments, urine was added at a N dose of 159 mg N/kg. The LDH-P dose was equivalent to 50 mg P/kg and sludge to 27 mg P/kg. Ryegrass was grown on treatments with and without nutrient compensation (Fig. 1).

The soil sample was sieved (3.9 mm) and air dried prior to mixing with fertilizers. The mineral N and P doses were added as stock solutions (50 g N/L as NH_4_NO_3_ and 20 g P/L as KH_2_PO_4_) and mixed into soil in 1.17 kg aliquots. The LDHs were added as powder (1.25 g/kg soil) and mixed into air dry soil followed by watering and mixing to equal final soil moisture content as the other treatments. Stored urine was added at 22.7 mL/ kg dry soil. Sewage sludge was obtained from the wastewater treatment and a mixture was made by mixing 250 mL of urine per kg of dry sewage sludge (fresh weight). The N concentration of the mixture was 10.1 g N/kg and the P concentration of the mix was 9.02 g P/kg. The mixture was added to the soil at 1.51 g/kg dry soil. Micronutrients and K were added to all pots in sufficient doses (200 mg K/kg dry soil as K_2_SO_4_, 40 mg Mg/kg dry soil as MgSO_4_ and 1 mg Fe/kg dry soil as FeSO_4_ 7H_2_O), to ensure there where no other nutrient deficiencies. Because P compensation was done using KH_2_PO_4_, the dosing of K was not equal in all treatments, but the soil available K was sufficiently large to cover all requirement. This was confirmed by foliar K analysis: all shoot K concentrations exceeded 4.5 g/kg dry weight, which is the highest reported concentrations for adequate supply^29^.

Ryegrass (*L. perenne*,) was sown in the pots (1 g/pot) and the plants were grown in a randomised set-up in a greenhouse between 17/05/2019 and 31/07/2019. Water loss was restored several times per week by weighing using deionised water and pot positions were regularly randomized. The ryegrass was cut at days 22, 42, 63 and 75 and dried at 40 °C for 5 days. After the final harvest, soil was sampled after removal of the majority of roots, the soil was air dried and stored pending analysis of E-values (see below) and acid ammonium oxalate extractable P^26^.

### Plant analysis

The oven dried samples of each replicate pot and harvest were powdered. Subsamples of 50 mg were digested on 10 mL tubes with 1 mL of 67–70% HNO_3_ and allowed to rest overnight prior to boiling for about 4 h to almost dryness, then diluted to 10 mL and analysed for elements by ICP-OES. Internal reference material was used to verify analytical accuracy. Total N was measured by dry combustion with an elemental analyzer (Thermo Scientific, EA1108) via combustion at 900 °C and subsequent analysis of CO_2_ and N_2_ with gas chromatography.

### E-value analysis

Isotopically exchangeable P concentration in soil was measured after the plant growth test according to Maertens et al.^30^. Only N- or P-added urine derived treatments were used as these are more relevant than their non-added counterparts. For every replicate, 3 g of mixed dried soil was added to 29 mL of 0.05 M CaCl_2_ solution. The suspension was shaken end-over-end for 16 h. Afterwards, 1 mL containing 40 kBq of ^32^P was added to the suspension and again shaken end-over-end for 48 h. Simultaneously, anion exchange membranes (AEM) were shaken end-over-end in 0.5 M NaHCO_3_ for 16 h and rinsed with ultrapure water. Then, the AEM were added to the spiked soils suspension and shaken end-over-end for 6 h. After this, the AEM were rinsed and put into 50 mL of 0.5 M HCl for 24 h. The activity and total P concentration in solution were measured by β-counter (Tricarb 3110 TR) and the malachite green colorimetric method respectively.

The E-value was calculated using the formula:

1 $$ E = \frac{{31_{P} }}{f}\;\;\;{\text{with}}\;\;\;f = \frac{{32_{{P_{t} }} }}{{32_{{P_{ini} }} }}. $$

where ^31^P is the concentration of P, ^32^P_t_ is the ^32^P activity at the time of measurement and ^32^P_ini_ is the activity of ^32^P at the moment of spiking.

### Statistical analyses

relative fertiliser PUE is defined here as the shoot P uptake in the urine derived fertilisers divided by the shoot P uptake at equivalent mineral P and N doses, the latter added through the soluble mineral fertilisers. Because an exact equivalent dosing was not possible, interpolation from the N, P-response surface was done. Cumulative shoot dry matter yield and cumulative shoot P uptake was calculated for every replicate pot of the 15 different treatments. The plant responses y (either yield or P uptake) to the N and P supplies in the nine mineral fertiliser treatments were fitted with surface plot as:

2 $$ y = \beta_{0} + \beta_{1} N_{input} + \beta_{2} P_{input} + \beta_{3} N_{input} P_{input} $$

where fertiliser input values (N_input_ and P_input_) are the nominal input values expressed as mg nutrient per kg air dry soil and β_0_, β_1_, β_2_ and β_3_ the parameters fitted by the model.

The relative fertiliser use efficiency of the urine derived fertilisers to mineral fertilisers was tested by adding the individual treatments, one by one, to the curve obtained for the mineral fertilisers and measuring the significant deviation by testing β_*4*_ > *0 or* β_*4*_ < *0* at significance levels of P < 0.05 and P < 0.01 in

3 $$ y = \beta_{0} + \beta_{1} N_{input} + \beta_{2} P_{input} + \beta_{3} N_{input} P_{input} + \beta_{4} Dummy. $$

where fertiliser input values (N_input_ and P_input_) are the nominal input values expressed as mg nutrient per kg air dry soil and β_0_, β_1_, β_2_ and β_3_ the parameters fitted by the model and Dummy = 0 for the mineral fertiliser treatments and Dummy = 1 for the urine derived treatments.

The same statistical procedure was used to determine significant differences in the sum of the total P uptake and the residual E-AEM value:

4 $$ y = \beta_{0} + \beta_{1} P_{input} + \beta_{2} Dummy $$

where fertiliser input values (N_input_ and P_input_) are the nominal input values expressed as mg nutrient per kg air dry soil and β_0_, β_1_, β_2_ and β_3_ the parameters fitted by the model and Dummy = 0 for the mineral fertiliser treatments and Dummy = 1 for the urine derived treatments.

As only the N-added treatments were used in this analysis, the terms containing the N input were omitted in this equation.

## Results and discussion

### Plant yield

The cumulative grass yield responded to N, P and its interaction with a markedly stronger effect of N addition than that of P addition (Table 1). The same model was fitted on the yields of the first cut and on cumulative yields of the second and third cuts (Table SI 1). After the first cut, only N addition significantly enhanced yield. This result indicates that P availability was initially not limiting plant growth. After the second cut and third cuts, the effect of P addition was significant, and the extent of the effect on yield due to P addition increased with increasing growth. From here onwards, P was thus limiting plant growth where insufficient P was added to the soil. Shoot analysis in the control soil at final harvest confirmed pronounced N deficiency (shoot N content < 0.23 g/kg), P deficiency (shoot P < 2.5 g/kg, except for the mineral 100 P 250 N treatment) while none of other nutrients (Mg, K, S, Ca, Fe and micronutrients) was at deficient levels when contrasting with deficiency thresholds. The shoot became yellow as a sign of N-deficiency in control treatment at already the second harvest while none of the shoots in the 250 mg N/kg soil with 100 mg P/kg soil treatments had visual signs of deficiency or toxicity.

**Table 1:** The shoot yield (g/pot) and P uptake (mg/pot) in ryegrass of the different mineral N and P treatments.

Mineral fertiliser	Yield (g/pot)	P uptake (mg/pot)
0 N 0 P	1.61 ± 0.02	7.4 ± 0.9
0 N 50 P	1.68 ± 0.24	10.9 ± 1.2
0 N 100 P	1.63 ± 0.03	10.8 ± 0.8
150 N 0 P	4.21 ± 0.30	10.7 ± 3.0
150 N 50 P	4.27 ± 1.00	19.0 ± 5.2
150 N 100 P	4.48 ± 0.05	19.6 ± 2.6
250 N 0 P	5.52 ± 0.97	12.3 ± 1.1
250 N 50 P	6.55 ± 0.32	22.7 ± 2.6
250 N 100 P	6.60 ± 0.22	23.3 ± 1.9
**Statistical effects of**^$^		
N	***	***
P	**	***
N × P	**	**

Data are means (± 95% confidence intervals) of the cumulative yield and cumulative P uptake of the four cuttings. Treatment codes show the dose (mg N or P/kg soil).

^$^**: *P* < 0.01; *** = *P* < 0.001.

All urine derived fertilisers enhanced cumulative yields compared to the unfertilised control soil. There were no significant differences in plant yield between the non-nutrient added urine derived treatments and the mineral treatments at equivalent N, P input (Table 2). The yield in these treatments was limited by the non-added nutrient, i.e. N for the LDH-P and the sludge mixed with urine treatments and P for the stored urine treatments. In contrast, the yield was lower in stored urine + P treatment and in the sludge/urine mixture + N than in the mineral treatments at equivalent N, P input (Table 2). The cumulative yield of the plants grown on stored urine + P started to be significantly lower than the mineral treatments from the third cut onwards (Table SI 2) at which the cumulative N uptake in the plants were significantly lower than those in plants grown on the most corresponding mineral treatments (168 g N/kg versus 186 g N/kg at final harvest). This indicates that urine derived total N has a lower availability than mineral N and that N is becoming a limiting nutrient from this point onwards. Shoot N content in the urine treatment were 17.7 g N/kg in the third cut, compared to 18.2 g N/kg predicted by the mineral treatments. A likely explanation is the volatilisation of N due to urea hydrolysis. While the hydrolysis of urea, the most abundant organic N molecule in urine, is slow in conditions where urease is inhibited, it is very fast when urease is present and active, such as in soils^31^. It is thus expected that urea hydrolysis is complete in the stored urine. The resulting ammonia can volatilise from the soil and thus negatively affect the yield of plants grown on urine as a N-source. Furthermore, other organic N molecules such as creatinine have a much more rigid structure and have as such much slower hydrolysis kinetics^32^. It is possible that the hydrolysis of these molecules is incomplete during the growing period and that the available N in the treatment is thus lower than the total N dosed, which reduces the yield of the treatment. The lower yield in the sludge/urine + N mixture than in mineral treatment is likely related to lower P availability in the sludge as will be discussed with the P uptake data.

**Table 2:** The shoot yield (g/pot) and P uptake (mg/pot) in ryegrass of the different urine derived fertilisers.

	Yield (g/pot)			P uptake (mg/pot)		
	Observed	Predicted	Effect	Observed	Predicted	Effect
Stored urine	4.49	4.25	n.s	11.8	12.4	n.s
Stored urine, P-added	**4.29**	4.81	*	**16.5**	21.0	*
LDH-P	1.92	1.60	n.s	8.4	10.0	n.s
LDH-P, N-added	6.56	6.17	n.s	19.8	19.8	n.s
Urine and sewage sludge	1.64	1.93	n.s	8.1	10.0	n.s
Urine and sewage sludge, N-added	**5.73**	6.26	*	**13.0**	17.8	**

Data are means (± 95% confidence intervals) of the cumulative yield and cumulative P uptake. The fertiliser codes and N, P doses are shown in Fig. 1, the predicted values of yield and P uptake refer to the prediction of the models based on the mineral treatments. The difference between predicted and observed values is indicated by the level of significance: n.s. = not significant, *: *p* < 0.05; ** = *p* < 0.01.

Bold values show lower performance of urine based fertilisers than the mineral fertilisers.

### P uptake

The total cumulative P uptake responded to N, P and its interaction with a markedly stronger effect of N addition than that of P addition (Table 1). The same model was fitted on the P uptake of the first, second and third cuts (Table SI 3 and SI 4). After the first cut, only P addition significantly enhanced P uptake. Hence, at the initial stage, the addition of N increased yield at an equal P uptake while the addition of P enhanced P uptake without increasing plant yield, i.e. suggesting luxury P uptake. From the second cut onwards, the cumulative P uptake is significant at the P < 0.01 significance level. Thus, N addition increased both plant yield and plant P uptake as P becomes a limiting nutrient. The N-P interaction becomes significant (P < 0.05) from the third cut onwards.

All urine derived fertilisers enhanced cumulative P uptake compared to the no fertilised control soil. There were no significant differences in the P uptake between the non-nutrient added urine fertiliser treatments and the mineral treatments (Tables 1 and 2). In contrast, the P uptake in the P-added stored urine treatment was 84% compared to corresponding mineral treatment. The P uptake of the N-added sludge mixed with urine treatments was only 57% of the corresponding mineral treatment (Table 1 and 2). In the P-added stored urine, the lower P uptake is likely an indirect N-effect on growth because shoot P content in that treatment was indicating adequate P availability. In contrast, shoot P content in soil treated with urine/sludge + N mixture was only 55% of the corresponding mineral treatment in the 4th cut. This shows that urine mixed with sludge has lower available P than conventional P fertilisers which negatively affects the yield of crops grown in these treatments.

The low PUE of plants grown on sewage sludges compared conventional fertiliser is attributed to the low solubility of the precipitated phosphates^33^. However, it should be noted that the P speciation in sewage sludge is often ill defined. It is highly dependent on the P removal method used and the post treatment of the sludge and can range from Fe- and Al phosphates to Ca phosphates and apatite like species^33,34^. For instance, a large study undertaken by Römer & Steingrobe^35^ using sandy and loamy soils showed that maize and winter wheat had a PUE of under 25% when grown on most treated sewage sludge ashes, with only Mg treated sewage sludge having a PUE higher than 50%. Nanzer et al.^36^ obtained similar results where plants grown on Mg-treated sewage sludge had higher yields and P uptake compared to Ca-treated sewage sludge in an acid soil. The difference is attributed to the higher solubility of Mg phosphate precipitates compared to Ca phosphates. These results were also confirmed long term field trials. Over 30 years, both N and P derived from sewage sludge had very low fertilisation potential^37^.

In contrast, P-loaded LDHs can have fertiliser efficiencies that are comparable to conventional fertilisers. The results are consistent with our research on P recovery from urine using LDHs. About 84% of the P loaded onto LDHs from synthetic urine for 24 h could be removed in a carbonate solution^20,38^. These high removal results already suggested a high potential use for fertilisation, which is now confirmed. Research has already been done to test the fertiliser potential of LDH-P. Everaert et al.^24^ found no differences in plant yield between powdered P-loaded LDH and a soluble P fertiliser (mono-ammonium phosphate) but they did report a large difference in yield for granulated fertilisers because of the slow release properties of LDHs. In another research, Everaert et al.^23^ showed that LDH-P improved P uptake in plants by 4.5 times in an acid soil. That effect was attributed to the liming effect of the alkaline LDHs. Similarly, Benício et al. (2016) found higher plant yields and P uptake in plants grown on LDH-P compared to plants grown on triple super phosphate. While LDH-P have potential to be used in both alkaline and acid soils they are expected to perform best in acid soils due to the aforementioned liming effect.

### Other elements: Na and Cd

Urine is a solution with a high ionic strength and dominated by Na. This is important to consider as long term application of urine derived fertilisers may results in soil salinity or sodicity in lower rainfall areas. Surface response curves were fitted to the mineral treatments and were used to identify elevated Na content in the plants. The Na contents (Tables SI 5 and 6) in the plants fertilised with stored urine and LDH-P were significantly (but only moderately) higher than those in the mineral treatments. The 3d cut of the LDH treatment had a Na content of 0.95 g Na/kg compared to the Na content of 1.01 g Na/kg in the relevant mineral treatment. The Na content of the 3rd cut of the P-added urine treatment was 2.34 g Na/kg compared to 0.97 g Na/kg for the corresponding mineral treatment. Ryegrass has a high tolerance for Na salt injury, as long as there is sufficient potassium (K) available for the plants^29^. The K content of the plants is above 4.5% in all of the treatments, which is the highest reported sufficient concentration^29^. Hence, it is unlikely that the elevated Na supply limited plant growth in the urine treatments. Sodicity could however be a problem when using urine as a fertiliser in soils where less K is added or when crops which are less tolerant for Na are grown on the soil.

Cadmium (Cd) is an important contaminant in the food chain and can be mobilised by complexation with chloride (Cl) present in urine. Cadmium contaminations of the food chain have been related to kidney functioning^25^. Urine contains between 1870 and 8400 mg Cl/L^39^. The urine dose here (25 mL/kg soil) is equivalent to about 89 mg Cl/kg soil. Crop Cd concentrations increase due to chloride, but only markedly above about 300 mg Cl/kg soil^40^. The shoot Cd content (Tables SI 7 and 8) at 250 mg N/kg soil with 100 mg P/kg soil input was 200 µg /kg and did generally not increase significantly with urine derived fertilisers at any harvest except for the Cd content in the 2^nd^ cut, where the shoot Cd content was 400 µg/kg in the 250 mg N/kg soil with 100 mg P/kg soil mineral treatment. A similar increase was found in the other treatments indicating that this increase is not related to the treatments. Possibly, water content in the soil was slightly lower during this growth period before harvest, which increases salinity and thus Cd uptake. This suggests that the dose of Cl was still moderate at the added urine doses in this experiment. While Cd uptake did not seem to be a problem in this experiment, it is however clear that repeated urine addition in low rainfall areas will aggravate the salinity stress and risk of Cd accumulation in the crop. This is especially so as Cd has a long biological half-live (15–20 years), meaning that chronic exposure is more important than acute exposure. In other words, long term exposure to moderately contaminated food is more important than a single consumption of high Cd containing food. Consistent solubilisation of Cd by Cl might thus be important over longer periods^25^.

### Residual value of soil P and fertiliser use efficiency of recycled P with LDHs

**Figure 2:**
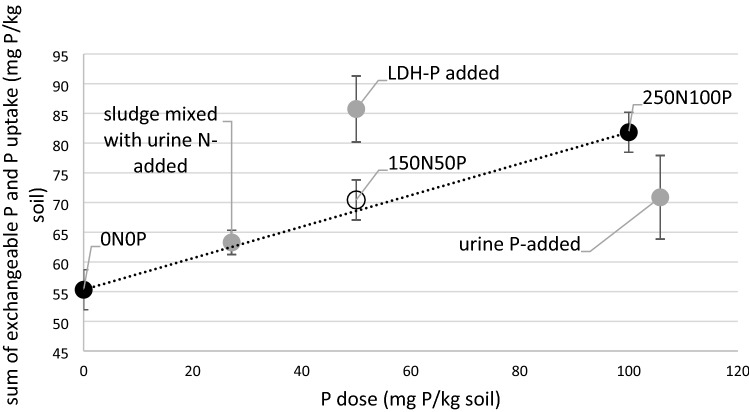
The sum of the isotopically exchangeable P after the pot trial and P uptake by plants during the pot trials in mg P/pot versus the P dose in mg P/pot. The error bars show the 95% confidence intervals. The 150N50P mineral fertiliser treatment was not measured but estimated assuming equal loss of P fixation per unit dose as at the 250N100P treatments. The sum is significantly higher for the LDH-P treatment compared to the linear response model (dashed line) for the soluble mineral fertilisers, indicating higher soil accessible P and lower loss by fixation in the LDH-P fertiliser.

The sum of the E-values after harvest and shoot P uptake is an indication of the total bioavailable P in the soil (Fig. 2). The urine and LDH-P treatments differed significantly from the linear response model based on the mineral treatments. Interestingly, there was no significant difference in the sum in the sludge mixed with urine treatment compared to the sum of the linear response model. This suggest that while the P uptake is lower, still significant amounts of residual P are left in the soil, potentially accessible in later growing seasons. Still, as the P uptake and, more importantly, the yield of these treatments are low, sludge is not recommended as a P fertiliser. The sum is lower for the urine treatment compared to the sum of linear response model, meaning urine is not an efficient P source for crops. Interestingly, the sum is significantly higher for the LDH treatment compared to the sum of the linear response model, the difference is related to a higher residual E-value in the LDH treatments. This result confirms the hypothesised slow release properties of LDH-P. The yield and the P uptake are comparable to soluble fertilisers over the growing period. However, the higher residual value suggest that more P remains available after the growing period, which could potentially be used in subsequent growing periods on more mature crops, thus enhancing the efficiency of LDH-P compared to soluble fertilisers. It is likely that other slow release P fertilisers such as struvite show similar increased residual E-values in the soil. An incubation experiment performed by Everaert et al.^24^ indeed shows that after a 100 day period more P can be recovered from the soil in struvite and LDH-P treatments compared to mono-ammonium phosphate, a soluble P fertiliser.

This research indicates that LDH-P have similar P efficiencies as conventional fertilisers, at least during growing times investigated in these experiments, confirming earlier work with LDHs by Everaert et al.^41^. Previously, slow release P fertiliser have been hypothesised to even have improved P efficiencies compared to conventional fertilisers^22,42^, this was not found here. Care should be given to the method of fertiliser application, as a pot trial performed by Everaert et al.^24^ suggests that granulated LDH-P or struvite might actually, at least in the first growing season, have lower fertiliser efficiencies compared to a granulated soluble P source, especially in highly weathered soils. In these type of soil, the liming effect of LDH-P might become important. Slow release fertilisers have however shown significantly lower runoff in rainfall simulation studies^43^, lowering P loss from the soil and decreasing eutrophication risks.

## Conclusion

The experiment assesses the applicability of urine derived fertilisers (urine, urine mixed with sludge and LDH-P) in an incubation test and a ryegrass pot trial. The incubation test suggests no differences in fertilisation efficiency of slow release P fertilisers compared to conventional soluble P fertilisers. Plants grown on urine and urine mixed with sludge have lower yield and P uptake compared to plants grown on a mineral fertiliser. This indicates that the P in urine mixed with sludge is less bioavailable than in conventional fertilisers and that N availability could be reducing yields when using urine as a fertiliser. In contrast, LDH-P performed well and yielded an equal biomass production with equal P uptake compared to a mineral fertiliser. The residual E-AEM values of LDH-P was furthermore higher than those of mineral treatments in the pot trial, suggesting a higher efficiency of LDH-P compared to conventional fertilisers over more or longer growing periods.

## Supplementary Information

Supplementary Information.

## References

[1] Dobermann A, George T, Thevs N (2002). Phosphorus fertilizer effects on soil phosphorus pools in acid upland soils. Soil Sci. Soc. Am. J..

[2] Lombi E, McLaughlin MJ, Johnston C, Armstrong RD, Holloway RE (2004). Mobility and lability of phosphorus from granular and fluid monoammonium phosphate differs in a calcareous soil. Soil Sci. Soc. Am. J..

[3] Chitrakar R (2006). Phosphate adsorption on synthetic goethite and akaganeite. J. Colloid Interface Sci..

[4] Withers PJA (2015). Greening the global phosphorus cycle: how green chemistry can help achieve planetary P sustainability. Green Chem..

[5] Schröder, J. J., Cordell, D., Smit, A. L. & Rosemarin, A. *Sustainable use of phosphorus*. *Plant Research International* http://www.susana.org/docs_ccbk/susana_download/2-1587-sustainableuseofphosphorusfinalsustpenvb120090025.pdf (2010).

[6] Cordell D, Drangert J-O, White S (2009). The story of phosphorus: global food security and food for thought. Glob. Environ. Chang..

[7] Ronteltap M (2009). Phosphorus recovery from source-separated urine through the precipitation of struvite.

[8] Morse GK, Brett SW, Guy JA, Lester JNU (1998). Review : Phosphorus removal and recovery technologies. Sci. Total Environ..

[9] Desmidt E (2015). Global phosphorus scarcity and full-scale P-recovery techniques: a review. Crit. Rev. Environ. Sci. Technol..

[10] de Kreuk MK, Heijnen JJ, van Loosdrecht MCM (2005). Simultaneous COD, nitrogen, and phosphate removal by aerobic granular sludge. Biotechnol. Bioeng..

[11] Vogel T, Nelles M, Eichler-Löbermann B (2015). Phosphorus application with recycled products from municipal waste water to different crop species. Ecol. Eng..

[12] Deeks LK (2013). A new sludge-derived organo-mineral fertilizer gives similar crop yields as conventional fertilizers. Agron. Sustain. Dev..

[13] Jaffer Y, Clark TA, Pearce P, Parsons SA (2002). Potential phosphorus recovery by struvite formation. Water Res..

[14] Doyle JD, Parsons SA (2002). Struvite formation, control and recovery. Water Res..

[15] Xu K (2015). The precipitation of magnesium potassium phosphate hexahydrate for P and K recovery from synthetic urine. Water Res..

[16] Xu K, Wang C, Liu H, Qian Y (2011). Simultaneous removal of phosphorus and potassium from synthetic urine through the precipitation of magnesium potassium phosphate hexahydrate. Chemosphere.

[17] Cabeza R, Steingrobe B, Römer W, Claassen N (2011). Effectiveness of recycled P products as P fertilizers, as evaluated in pot experiments. Nutr. Cycl. Agroecosyst..

[18] Antonini S, Arias MA, Eichert T, Clemens J (2012). Greenhouse evaluation and environmental impact assessment of different urine-derived struvite fertilizers as phosphorus sources for plants. Chemosphere.

[19] Talboys PJ (2016). Struvite: a slow-release fertiliser for sustainable phosphorus management?. Plant Soil.

[20] Dox K, Everaert M, Merckx R, Smolders E (2019). Optimization of phosphate recovery from urine by layered double hydroxides. Sci. Total Environ..

[21] Evans DG, Slade RCT (2006). Structural aspects of layered double hydroxides. Struct. Bond..

[22] Benício LPF (2016). Layered double hydroxides: new technology in phosphate fertilizers based on nanostructured materials. ACS Sustain. Chem. Eng..

[23] Everaert M (2016). Phosphate-exchanged Mg–Al layered double hydroxides: a new slow release phosphate fertilizer. ACS Sustain. Chem. Eng..

[24] Everaert M, Degryse F, McLaughlin MJ, de Vos D, Smolders E (2017). Agronomic effectiveness of granulated and powdered P-exchanged Mg–Al LDH relative to struvite and MAP. J. Agric. Food Chem..

[25] Smolders, E. & Mertens, J. Chapter 10: Cadmium. in *Heavy metals in soils* 283–311 (2013). 10.1016/s0165-9936(96)90032-1.

[26] Nawara S (2017). A comparison of soil tests for available phosphorus in long-term field experiments in Europe. Eur. J. Soil Sci..

[27] He J (2006). Preparation of layered double hydroxides. Struct. Bond..

[28] Griffith DP, Musher DM, Itin C (1976). Urease: the primary cause of infection-induced urinary stones. Invest. Urol..

[29] Reuter DJ, Robinson JB (1997). Plant analysis an interpretation manual.

[30] Maertens E (2004). An anion resin membrane technique to overcome detection limits of isotopically exchanged P in P-sorbing soils. Eur. J. Soil Sci..

[31] Sigurdarson JJ, Svane S, Karring H (2018). The molecular processes of urea hydrolysis in relation to ammonia emissions from agriculture. Rev. Environ. Sci. Biotechnol..

[32] Miller TJ (1991). The kinetics and mechanism of the hydrolysis of creatinine in urine. Anal. Lett..

[33] Wilfert P, Kumar PS, Korving L, Witkamp GJ, van Loosdrecht MCM (2015). The relevance of phosphorus and iron chemistry to the recovery of phosphorus from wastewater: a review. Environ. Sci. Technol..

[34] Nanzer S, Oberson A, Huthwelker T, Eggenberger U, Frossard E (2014). The molecular environment of phosphorus in sewage sludge ash: implications for bioavailability. J. Environ. Qual..

[35] Römer W, Steingrobe B (2018). Fertilizer effect of phosphorus recycling products. Sustainability (Switzerland).

[36] Nanzer S, Oberson A, Eggenberger U, Frossard E (2019). Predicting phosphate release from sewage sludge ash using an ion sink assay. J. Environ. Qual..

[37] Börjesson G, Kätterer T (2018). Soil fertility effects of repeated application of sewage sludge in two 30-year-old field experiments. Nutr. Cycl. Agroecosyst..

[38] Dox K, Pareijn R, Everaert M, Smolders E (2019). Phosphorus recycling from urine using layered double hydroxides: a kinetic study. Appl. Clay Sci..

[39] Putnam, D. *Composition and Concentrative Properties Of Human Urine*. (1971).

[40] McLaughlin MJ, Palmer LT, Tiller KG, Beech TA, Smart MK (1994). Increased soil salinity causes elevated cadmium concentrations in field-grown potato tubers. J. Environ. Qual..

[41] Everaert M (2018). The isotopic exchangeability of phosphate in Mg–Al layered double hydroxides. J. Colloid Interface Sci..

[42] Shaviv A (2000). Advances in controlled release of fertilizers. Adv. Agron..

[43] Everaert M, da Silva RC, Degryse F, McLaughlin MJ, Smolders E (2018). Limited dissolved phosphorus runoff losses from layered double hydroxide and struvite fertilizers in a rainfall simulation study. J. Environ. Qual..

