# Supplementary material for: Superior residual fertiliser value in soil with phosphorus recycled from urine in layered double hydroxides
Source: Sci Rep. 2022 May 16;12:8092. doi: 10.1038/s41598-022-11892-4 (PMC9110350; doi:10.1038/s41598-022-11892-4)
Supplement: Supplementary file 1 — Supplementary Information. [file 41598_2022_11892_MOESM1_ESM.docx]

Table SI 1. The cumulative shoot yield (g/pot) in ryegrass of the different mineral N and P treatments. Data are means (± 95% confidence intervals) of the cumulative yield in of the four cuttings. Treatment codes show the dose (mg N or P/kg soil).

| Mineral fertiliser | Yield 1^st^ cut (g/pot) | Yield 2^nd^ cut (g/pot) | Yield 3^rd^ cut (g/pot) |
| --- | --- | --- | --- |
|  |  |  |  |
| 0 N 0 P | 0.90 ± 0.02 | 1.27 ± 0.08 | 1.48 ± 0.04 |
| 0 N 50 P | 0.94 ± 0.12 | 1.34 ± 0.10 | 1.57 ± 0.23 |
| 0 N 100 P | 0.96 ± 0.09 | 1.30 ± 0.06 | 1.51 ± 0.08 |
| 150 N 0 P | 1.25 ± 0.28 | 3.06 ± 0.76 | 3.94 ± 0.34 |
| 150 N 50 P | 1.42 ± 0.31 | 2.94 ± 0.96 | 3.78 ± 1.05 |
| 150 N 100 P | 1.45 ± 0.23 | 3.52 ± 0.32 | 4.22 ± 0.12 |
| 250 N 0 P | 1.23 ± 0.36 | 2.95 ± 0.31 | 4.91 ± 0.91 |
| 250 N 50 P | 1.41 ± 0.18 | 3.95 ± 0.22 | 6.02 ± 0.35 |
| 250 N 100 P | 1.41 ± 0.40 | 3.78 ± 0.59 | 6.02 ± 0.17 |
| statistical effects of^$^ |  |  |  |
| N | *** | *** | *** |
| P | n.s. | * | *** |
| N x P | n.s. | n.s. | *** |

$*: *P* < 0.05**; *P* < 0.01; ***= *P*<0.001.

Table SI 2. The shoot yield (g/pot) in ryegrass of the different urine derived fertilisers. Data are means (± 95% confidence intervals) of the cumulative yield in of the first three cuttings. The fertiliser codes and N, P doses are shown in Figure 1, the predicted values of yield and P uptake refer to the prediction of the models based on the mineral treatments. The difference between predicted and observed values is indicated by the level of significance: n.s.=not significant, *: p<0.05; **= p<0.01.

|  | Yield 1^st^ cut (g/pot) | | |  | Yield 2^nd^ cut (g/pot) | | |  | Yield 3^rd^ cut (g/pot) | | |
| --- | --- | --- | --- | --- | --- | --- | --- | --- | --- | --- | --- |
|  | observed | predicted | effect |  | observed | predicted | effect |  | observed | predicted | effect |
| stored urine | 1.41 | 1.18 | * |  | 3.25 | 2.69 | * |  | 4.15 | 3.88 | n.s. |
| stored urine, P-added | 1.45 | 1.34 | n.s. |  | 3.46 | 3.20 | n.s. |  | 4.04 | 4.46 | * |
| LDH-P | 0.94 | 0.99 | n.s. |  | 1.48 | 1.44 | n.s. |  | 1.74 | 1.52 | n.s. |
| LDH-P, N-added | 1.19 | 1.41 | * |  | 3.45 | 3.76 | n.s. |  | 5.91 | 5.64 | n.s. |
| urine and sewage sludge | 0.81 | 1.00 | n.s. |  | 1.24 | 1.59 | n.s. |  | 1.47 | 1.81 | n.s. |
| urine and sewage sludge, N-added | 1.07 | 1.39 | ** |  | 2.85 | 3.73 | ** |  | 5.03 | 5.70 | ** |

Table SI 3. The cumulative P uptake (g/pot) in ryegrass of the different mineral N and P treatments. Data are means (± 95% confidence intervals) of the cumulative P uptake in of the four cuttings. Treatment codes show the dose (mg N or P/kg soil).

| Mineral fertiliser | P uptake 1^st^ cut (g/pot) | P uptake 2^nd^ cut (g/pot) | P uptake 3^rd^ cut (g/pot) |
| --- | --- | --- | --- |
|  |  |  |  |
| 0 N 0 P | 3.47 ± 0.18 | 5.49 ± 0.93 | 6.64 ± 0.67 |
| 0 N 50 P | 5.87 ± 0.79 | 8.71 ± 0.86 | 10.19 ± 1.42 |
| 0 N 100 P | 6.20 ± 0.61 | 8.48 ± 0.77 | 9.99 ± 1.18 |
| 150 N 0 P | 3.33 ± 0.45 | 7.05 ± 2.83 | 9.34 ± 3.11 |
| 150 N 50 P | 6.11 ± 0.50 | 12.33 ± 3.14 | 17.47 ± 4.69 |
| 150 N 100 P | 6.24 ± 1.10 | 13.43 ± 2.30 | 18.06 ± 2.36 |
| 250 N 0 P | 3.05 ± 0.72 | 6.28 ± 1.46 | 9.99 ± 1.44 |
| 250 N 50 P | 5.32 ± 1.05 | 12.39 ± 2.15 | 19.27 ± 2.85 |
| 250 N 100 P | 5.69 ± 1.39 | 12.85 ± 1.06 | 19.66 ± 0.72 |
| statistical effects of^$^ |  |  |  |
| N | n.s. | ** | *** |
| P | *** | *** | *** |
| N x P | n.s. | * | *** |

$*: *P* < 0.05**; *P* < 0.01; ***= *P*<0.001.

Table SI 4. The cumulative P uptake (g/pot) in ryegrass of the different urine derived fertilisers. Data are means (± 95% confidence intervals) of the cumulative P uptake in of the first three cuttings. The fertiliser codes and N, P doses are shown in Figure 1, the predicted values of yield and P uptake refer to the prediction of the models based on the mineral treatments. The difference between predicted and observed values is indicated by the level of significance: n.s.=not significant, *: p<0.05; **= p<0.01.

|  | P uptake 1^st^ cut (g/pot) | | |  | P uptake 2^nd^ cut (g/pot) | | |  | P uptake 3^rd^ cut (g/pot) | | |
| --- | --- | --- | --- | --- | --- | --- | --- | --- | --- | --- | --- |
|  | observed | predicted | effect |  | observed | predicted | effect |  | observed | predicted | effect |
| stored urine | 3.69 | 3.76 | n.s. |  | 7.25 | 7.47 | n.s. |  | 10.19 | 10.68 | n.s. |
| stored urine, P-added | 5.71 | 6.53 | * |  | 11.84 | 13.16 | n.s. |  | 15.14 | 18.58 | * |
| LDH-P | 4.00 | 5.27 | ** |  | 6.18 | 7.98 | ** |  | 7.54 | 9.36 | * |
| LDH-P, N-added | 4.53 | 4.82 | n.s. |  | 10.56 | 11.14 | n.s. |  | 16.43 | 16.94 | n.s. |
| urine and sewage sludge | 3.62 | 4.60 | * |  | 5.79 | 7.39 | n.s. |  | 7.08 | 8.93 | n.s. |
| urine and sewage sludge, N-added | 3.00 | 4.16 | * |  | 6.48 | 9.69 | ** |  | 10.52 | 15.02 | ** |

Table SI 5. The Na content (mg/kg) (± 95% confidence intervals) in ryegrass of the different mineral N and P treatments. Treatment codes show the dose (mg N or P/kg soil).

| Mineral fertiliser | Na content 1^st^ cut (g/kg) | Na content 2^st^ cut (g/kg) | Na content 3^st^ cut (g/kg) |
| --- | --- | --- | --- |
|  |  |  |  |
| 0 N 0 P | 0.90 ± 0.58 | 0.77 ± 0.35 | 1.00 ± 0.35 |
| 0 N 50 P | 0.85 ± 0.38 | 0.71 ± 0.24 | 1.06 ± 0.22 |
| 0 N 100 P | 0.74 ± 0.10 | 0.65 ± 0.13 | 0.98 ± 0.18 |
| 150 N 0 P | 1.02 ± 0.48 | 0.97 ± 1.24 | 0.80 ± 0.20 |
| 150 N 50 P | 0.87 ± 0.75 | 0.78 ± 0.14 | 0.80 ± 0.11 |
| 150 N 100 P | 0.79 ± 0.20 | 0.82 ± 0.14 | 0.97 ± 0.09 |
| 250 N 0 P | 0.97 ± 0.08 | 0.76 ± 0.22 | 0.97 ± 0.24 |
| 250 N 50 P | 0.90 ± 0.14 | 0.81 ± 0.05 | 0.90 ± 0.03 |
| 250 N 100 P | 0.83 ± 0.09 | 0.90 ± 0.11 | 1.01 ± 0.17 |

Table SI 6. The Na content (mg/kg) (± 95% confidence intervals) in ryegrass of the different urine derived fertilisers. Treatment codes show the dose (mg N or P/kg soil).

| Mineral fertiliser | Na content 1^st^ cut (g/kg) | Na content 2^st^ cut (g/kg) | Na content 3^st^ cut (g/kg) |
| --- | --- | --- | --- |
|  |  |  |  |
| stored urine | 1.67 ± 0.14 | 1.53 ± 0.12 | 2.24 ± 0.98 |
| stored urine, P-added | 1.81 ± 0.35 | 1.67 ± 0.18 | 2.34 ± 0.28 |
| LDH-P | 1.48 ± 0.12 | 1.20 ± 0.10 | 1.30 ± 0.20 |
| LDH-P, N-added | 1.26 ± 0.02 | 1.06 ± 0.67 | 0.95 ± 0.30 |
| urine and sewage sludge | 1.04 ± 0.03 | 0.82 ± 0.07 | 0.99 ± 0.22 |
| urine and sewage sludge, N-added | 4.56 ± 0.08 | 0.86 ± 0.29 | 0.87 ± 0.27 |

Table SI 7. The Cd content (mg/kg) (± 95% confidence intervals) in ryegrass of the different mineral N and P treatments. Treatment codes show the dose (mg N or P/kg soil).

| Mineral fertiliser | Cd content 1^st^ cut (mg/kg) | Cd content 2^st^ cut (mg/kg) | Cd content 3^st^ cut (mg/kg) |
| --- | --- | --- | --- |
|  |  |  |  |
| 0 N 0 P | 0.25 ± 1.60 | 0.52 ± 0.43 | 0.23 ± 0.04 |
| 0 N 50 P | 0.33 ± 0.66 | 0.78 ± 0.35 | 0.18 ± 0.09 |
| 0 N 100 P | 0.22 ± 0.22 | 0.38 ± 0.71 | 0.30 ± 1.86 |
| 150 N 0 P | 0.30 ± 0.04 | 0.29 ± 0.07 | 0.19 ± 0.15 |
| 150 N 50 P | 0.34 ± 0.10 | 0.32 ± 0.39 | 0.17 ± 0.08 |
| 150 N 100 P | 0.31 ± 0.12 | 0.25 ± 0.05 | 0.17 ± 0.03 |
| 250 N 0 P | 0.46 ± 0.15 | 0.54 ± 0.33 | 0.31 ± 0.09 |
| 250 N 50 P | 0.33 ± 0.06 | 0.54 ± 0.50 | 0.25 ± 0.11 |
| 250 N 100 P | 0.30 ± 0.02 | 0.40 ± 0.25 | 0.20 ± 0.07 |

Table SI 8. The Cd content (mg/kg) (± 95% confidence intervals) in ryegrass of the different urine derived fertilisers.

| Mineral fertiliser | Cd content 1^st^ cut (mg/kg) | Cd content 2^st^ cut (mg/kg) | Cd content 3^st^ cut (mg/kg) |
| --- | --- | --- | --- |
|  |  |  |  |
| stored urine | 0.48 ± 0.22 | 0.74 ± 0.58 | 0.38 ± 0.11 |
| stored urine, P-added | 0.49 ± 0.34 | 0.51 ± 0.39 | 0.29 ± 0.29 |
| LDH-P | 0.42 ± 0.14 | 0.59 ± 0.81 | 0.36 ± 0.47 |
| LDH-P, N-added | 0.61 ± 0.74 | 0.46 ± 0.11 | 0.30 ± 0.29 |
| urine and sewage sludge | 0.49 ± 0.52 | 0.61 ± 0.75 | 0.14 ± 0.06 |
| urine and sewage sludge, N-added | 0.85 ± 0.47 | 0.72 ± 0.84 | 0.30 ± 0.19 |
